# Risk factors for spontaneous preterm birth among healthy nulliparous pregnant women in the Netherlands, a prospective cohort study

**DOI:** 10.1002/hsr2.585

**Published:** 2022-05-24

**Authors:** Heleen J. Schuster, Myrthe J. C. S. Peelen, Petra J. Hajenius, Monique D. M. van Beukering, Rik van Eekelen, Marit Schonewille, Henna Playfair, Joris A. M. van der Post, Marjolein Kok, Rebecca C. Painter

**Affiliations:** ^1^ Department of Obstetrics and Gynecology Amsterdam UMC location University of Amsterdam Amsterdam The Netherlands; ^2^ Amsterdam Reproduction and Development Amsterdam The Netherlands; ^3^ Department of Medical Microbiology and Infection Control Amsterdam UMC location University of Amsterdam Amsterdam The Netherlands; ^4^ Amsterdam Institute for Infection and Immunity Amsterdam The Netherlands; ^5^ Centre for Reproductive Medicine Amsterdam UMC location University of Amsterdam Amsterdam The Netherlands; ^6^ Midwifery Practice Bijlmermeer Amsterdam The Netherlands; ^7^ Department of Obstetrics and Gynecology Amsterdam UMC location Vrije Universiteit Amsterdam The Netherlands

**Keywords:** bio‐samples, cohort profile, nulliparous women, risk factors, spontaneous preterm birth

## Abstract

**Introduction:**

Spontaneous preterm birth (sPTB) is a major contributor to neonatal morbidity and mortality worldwide. The pathophysiology of sPTB is poorly understood, in particular among nulliparous women without apparent medical or obstetric risk factors. Therefore, we aimed to identify risk factors for sPTB in healthy nulliparous women.

**Material and Methods:**

We performed a prospective cohort study. Recruitment took place from February 2014 to December 2016 in 16 community midwifery centers in the Netherlands. Eligibility criteria were: ≥18 years, no previous pregnancy >16 weeks of gestation, healthy singleton pregnancy, and antenatal booking <24 weeks of gestation. At study inclusion, participants completed a questionnaire, including details on lifestyle, work, and medical history. Cervical length was measured by vaginal ultrasound at the second‐trimester anomaly scan. Detailed information concerning pregnancy and birth was collected via antenatal charts. We calculated the adjusted odds ratio (aOR) and 95% confidence intervals (CI) for various risk factors with correction for socioeconomic status (SES) using logistic regression and Firth's correction.

**Results:**

We included 363 women of whom pregnancy outcomes were available in 349 (96.1%) participants. The cervical length measurement was available for 225 (62.0%) participants. sPTB occurred in 26 women (7.5%). SES was associated with sPTB (OR: 3.7, 95%  CI: 1.6–8.5) in univariate analysis. First or second trimester vaginal bleeding (aOR: 3.6, 95% CI: 1.4–9.0) and urinary tract infection during pregnancy (aOR: 4.9, 95% CI: 1.7–13.9) were associated with sPTB in multivariate analysis.

**Conclusions:**

This prospective cohort confirms established risk factors for sPTB in nulliparous women deemed at low risk of sPTB.

## 1 INTRODUCTION

Preterm birth (PTB) is a major contributor to neonatal morbidity and mortality. Fifty to seventy percent of all neonatal mortality is attributable to PTB and strongly dependent on gestational age at birth.^1^, ^2^ Long‐term sequelae of PTB include cerebral palsy, visual and hearing impairment, chronic lung disease, behavioral problems, and intellectual impairment during childhood and adolescence.^3^, ^4^ Although various risk factors for spontaneous PTB (sPTB) are known, the pathophysiology is still not well understood.

Several interventions are available to prevent sPTB, but only target women at high risk based on their obstetric history (e.g., previous sPTB) or short cervical length at second‐trimester anomaly scan.^5^ In the Netherlands, 53.8% of the 11.705 PTBs in 2017 occurred in nulliparous women, who were not identified with available screening methods.^6^ Targeted preventive interventions for these nulliparous women cannot be developed or deployed without timely identification.

Various prediction models to identify women at risk for sPTB have been published.^7^, ^8^, ^9^, ^10^ A systematic review shows that published prediction models for sPTB failed to adequately identify women at risk, using only clinical risk factors such as previous PTB, ethnicity, and maternal age.^11^ A subgroup analysis of 1284 nulliparous women revealed poor discriminative performance, with discrimination C‐statistic ranging from 0.51 (95% CI: 0.45–0.57) to 0.55 (95% CI: 0.49–0.60). This systematic review shows the need for better identification of nulliparous women at high risk for sPTB.

We aimed to identify risk factors for sPTB among nulliparous women with a healthy pregnancy in a prospective multicenter cohort study in the Netherlands; the PRrevention Of PrEterm Labor in LOw Risk women study (the PROPELLOR study). In this study, we provided three questionnaires on lifestyle, work, and medical history, collected bio‐samples, measured cervical length during the routine abdominal second‐trimester anomaly scan, and collected data on pregnancy and birth. In this manuscript, we focus on clinical risk factors for sPTB.

## 2 MATERIALS AND METHODS

The PROPELLOR study was an observational cohort study, conducted in the catchment area of the Regional Perinatal Network North‐West Netherlands. STROBE reporting guidelines for cohort studies were used for this manuscript (Appendix S1). Recruitment took place between February 2014 and December 2016 in 16 midwifery practices. All nulliparous women ≥18 years, who booked for antenatal care in the participating midwifery practices before 24 weeks of gestation, and had a healthy singleton pregnancy at booking were eligible for inclusion in the study. Nulliparity was defined as never having had a pregnancy duration beyond 16 weeks of gestation.^12^ Healthy pregnancies were primarily women without any medical or surgical history, but could include women with a history of an uncomplicated loop excision of the cervix for cervical dysplasia, mild endocrine disorders (e.g., well‐controlled hypothyroidism), Class I or II obesity or mild psychiatric disease (well‐controlled depression, regardless of medication).^13^ Women with a history of late second‐trimester miscarriage >16 weeks of gestation were not eligible for this study as in these women the risk for subsequent second‐trimester miscarriage and PTB is significantly increased.^14^, ^15^ Other exclusion criteria were the inability to provide unassisted informed consent due to language or literacy issues and high‐risk pregnancies. High‐risk pregnancies were defined according to the guidelines of the Royal Dutch Organization of Midwives and included pregnancies with any underlying medical or endocrine condition (e.g., pregestational diabetes or hyperthyroidism), Class III obesity, history of uterine surgery or cervical cone biopsy, significant psychiatric disease (psychosis, bipolar disorder) or large uterine fibroids.

At the first prenatal visit, usually between 8 and 12 weeks of gestation, women were informed by their midwife about the study and asked to participate. Written informed consent was obtained from each participant.

The participants were asked to fill in three questionnaires, one in each trimester (Appendix S2). The first questionnaire was provided at inclusion and included socio‐demographics, general health, current pregnancy, obstetric history, obstetric family history, current working conditions, leisure, household characteristics, and possible domestic violence. The second and third questionnaires were provided at later gestational ages. Bio‐samples (blood sample and vaginal swab) were collected in the first trimester. For more details regarding the methods, including the questionnaires and bio‐samples, see Appendix S3.

Gestational age was determined by measurement of the fetal crown‐rump length at the first‐trimester ultrasound. Cervical length in millimeter (mm) was measured transvaginally during the routine abdominal second‐trimester anomaly scan carried out around 18–20 weeks of gestation. All ultrasound technicians performing the second‐trimester anomaly scan received training to achieve uniform cervical length measurement techniques in all participating practices. Standardized cervical measurement of the cervical length was based on previous research.^16^ Cervical length ≤25 mm measured during the second‐trimester anomaly ultrasound scan was considered to pose an increased risk for sPTB.^17^

For data on pregnancy and birth, we collected participants' antenatal files retrospectively via the midwifery practices or the relevant health care provider. All data presented originated from the antenatal files, except ethnicity and education level, which were primarily extracted from the first questionnaire. Ethnicity was based on participant self‐identification. The following categories were available: white‐European, African, Indian, Moroccan, Turkish, Middle Eastern, Asian, other Western, other non‐Western, and mixed. If the first questionnaire was not available, ethnicity, as reported in the antenatal file, was used.

The primary outcome measure was sPTB, defined as the spontaneous onset of labor or spontaneous preterm rupture of membranes, with discrimination between PTB between 23 and 37 weeks of gestation and late second‐trimester miscarriage between 16 and 22 weeks of gestation.

We used the CROWN initiative core outcome set for the reporting of secondary outcomes.^18^ In our study, the outcome “offspring infection” was defined as all cases of suspected early sepsis <72 h after birth and the outcome “respiratory morbidity” as all neonates requiring respiratory support. The outcomes “harm to mother and harm to offspring from intervention” were not reported as these were deemed not relevant due to the observational character of our study.

Medically indicated PTB was defined as delivery <37 weeks of gestation with induction of labor or primary cesarean section for maternal or fetal indications.

Pregnancy‐induced hypertension was defined as a systolic blood pressure ≥140 mmHg and/or a diastolic blood pressure of ≥90 mmHg in the absence of proteinuria measured on two separate occasions at least 6 h apart.^19^ Preeclampsia was defined as hypertension with proteinuria of ≥300 mg/24 h in a 24 h urine sample, according to the guidelines at the time the study was conducted.^19^ Gestational diabetes was diagnosed by a 2‐point 75‐g oral glucose tolerance test, with fasting capillary whole blood glucose ≥6.1 mmol/L or 2‐h postload capillary whole blood glucose ≥7.8 mmol/L.^20^ Small for gestational age was defined as birth weight less than the 10th percentile of the gestational age, based on the Netherlands Perinatal Registry reference weights which are available for pregnancies that end ≥23 weeks.^21^, ^22^

Perinatal mortality was defined as fetal antepartum or intrapartum death ≥22 weeks of gestation and postpartum death until 28 days after birth, according to guidelines by the Dutch Obstetrics and Gynecology Association.^23^

Participants could opt for a home or a hospital midwifery‐led delivery if their pregnancy and delivery are expected to be uneventful. If risk factors had arisen during pregnancy or labor, the delivery took place in the hospital in obstetrician‐led care. The maternity care system in The Netherlands is extensively described elsewhere.^24^

Socioeconomic status (SES) was estimated based on the postal code of residence and the status scores from the Netherlands Institute for Social Research. These SES scores were based on the average income in a postal code area, the number of inhabitants with a low educational status, the number of inhabitants with a low income, and the percentage of unemployment. Three categories for SES were defined as low (<20th percentile), middle (20th–80th percentile), and high (>80th percentile).^9^, ^25^, ^26^

Assisted reproductive technologies (ART) were intrauterine insemination, ovulation induction, in vitro fertilization, intracytoplasmic sperm injection, and gamete donation.

### 2.1 Statistical analyses

Baseline characteristics were presented as mean and standard deviation (SD) median and interquartile ranges (IQRs) or absolute numbers (*n*) and percentages based on the data before imputation. We handled missing baseline characteristics data using multiple imputations for the calculation of ORs. Numerical results are based on pooled estimates over 10 imputation sets using Rubin's rules.^27^

We performed univariate logistic regression for each risk factor for women with sPTB, calculating the odds ratio (OR) and 95% confidence intervals (CIs). The reference group consisted of all other participants in the study with available pregnancy outcomes, including medically indicated PTB. There could be overfitting in the model due to a limited number of events. In a sensitivity analysis, we refitted the significant associations using logistic regression with Firth's correction.^28^ Firth's correction uses penalized likelihood which aims to reduce the influence of a low number of events in overfitting, that is, overestimations of associations. Categorical and continuous variables were converted into binary variables wherever possible. In the analyses, the categories white‐European and other Western were combined in the group white‐European. A *p* < 0.05 was considered statistically significant. The limited number of cases of sPTB allowed correction for one confounder at a time. For risk factors with a *p* < 0.05, ORs were adjusted for SES (low or middle/high). The statistical software packages IBM SPSS Statistics 22 and 25 (Statistical Package for the Social Sciences, SPSS Inc.) and R version 3.3.2 (R Core Team, 2016) with the *mice*, *micepool*, and *logistf* packages were used.

For sample size calculation, see Appendix S3.

### 2.2 Patient and public involvement

The Regional Perinatal Network Northwest Netherlands, in which perinatal health care professions and patient representatives are represented, was involved in the study design and execution.

### 2.3 Ethical approval

The study was approved by the Medical Ethics Committee of the Amsterdam Medical Centre (now part of the Amsterdam University Medical Centers). The study received ethical clearance through the institutional review board (registration number NL43414.018.13).

## 3 RESULTS

**Figure 1:**
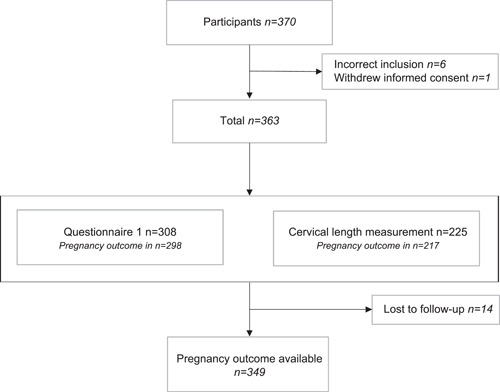
Flowchart PROPELLOR study

In total, 370 women consented to participate in the study. Six patients were later found to be ineligible and one withdrew informed consent. A total of 363 women were enrolled in the study. The cervical length measurement was available for 225 (62.0%) women and pregnancy outcomes for 349 (96.1%) women (Figure 1). Fourteen women (3.9%) were lost to follow‐up due to moving to an unknown address. For more details regarding all completed questionnaires and available bio‐samples, see Figure S1.

The baseline characteristics of the 363 study participants are presented in Table 1. The median gestational age at study entry was 10 weeks (IQR: 9.0–12.0). The majority self‐identified as white‐European (63.4%). One‐third of the participants (33.3%) had a low SES.

**Table 1:** Baseline characteristics PROPELLOR cohort

Baseline characteristics			Missing	
Total = 363	*n*	%	*n*	%
Primigravida	268	73.8	0	‐
Maternal age at inclusion, mean–SD	28.8	4.4	0	‐
Gestational age at inclusion (weeks), median–IQR	10	9.0–12.0	0	‐
Ethnicity	‐	‐	48	13.2
White‐European	230	63.4	‐	‐
African	33	9.1	‐	‐
Indian	8	2.2	‐	‐
Turkish	3	0.8	‐	‐
Middle Eastern	3	0.8	‐	‐
Asian	5	1.4	‐	‐
Mixed	20	5.5	‐	‐
Other non‐Western	13	3.6	‐	‐
Marital status	‐	‐	35	9.6
Married/cohabiting with partner	301	82.9	‐	‐
Relation, not living together	13	3.6	‐	‐
Single	14	3.9	‐	‐
Socioeconomic status	‐	‐	1	0.3
Low (<p20)	121	33.3	‐	‐
Middle (p20–80)	168	46.3	‐	‐
High (>p80)	73	20.1	‐	‐
University or higher vocational education	183	50.4	55	15.2
BMI (kg/m^2^), mean (SD)	23.8	4.3	3	0.9
Smoking	‐	‐	17	4.7
No	269	74.1	‐	‐
Yes	12	3.3	‐	‐
Quit preconception	11	3.0	‐	‐
Quit during pregnancy	54	14.9	‐	‐
Medical history	‐	‐	0	‐
No previous diagnosis	256	70.5	‐	‐
Asthma	15	4.1	‐	‐
Thyroid disease	9	2.5	‐	‐
Psychiatric history	15	4.1	0	‐
Previous cervical surgery^a^	32	8.8	14	3.9
Conception	‐	‐	1	0.3
Spontaneous	336	92.6	‐	‐
ART	26	7.2	‐	‐

*Note*: This table includes data on all study participants. Psychiatric history includes women with current or previous signs of depression and other psychiatric disorders.

Abbreviations: ART, assisted reproduction technologies; BMI, body mass index; IQR, interquartile range.

^a^ Previous cervical surgery includes history of pregnancy termination using dilation and curettage or loop excision of the cervix.

Pregnancy characteristics and outcomes are presented in Table 2. sPTB occurred in 26 (7.5%) participants, in 20 (5.7%) between 23 and 37 weeks of gestation, and in 6 (1.7%) between 16 and 22 weeks of gestation. Seven (2.0%) participants had a medically indicated PTB, primarily because of preeclampsia. Four participants (1.1%) had a first‐trimester miscarriage <16 weeks of gestation, one pregnancy (0.3%) was terminated because of congenital abnormalities and stillbirth occurred in one pregnancy (0.3%). Five pregnancies (1.4%) ended in perinatal mortality. Four neonates died of complications due to prematurity and one neonate delivered at term died within 24 h of birth because of a subarachnoid hemorrhage.

**Table 2:** Primary and core outcomes

Outcomes and pregnancy characteristics			Missing	
Total = 349	*n*	%	*n*	%
*Primary outcome*				
Spontaneous preterm birth (16–37 weeks)	26	7.5	0	‐
Between 23 and 37 weeks	20	5.7	‐	‐
Between 16 and 22 weeks	6	1.7	‐	‐
*CROWN initiative core outcomes*				
Maternal mortality (yes)	0	‐	20	5.5
Maternal infection or inflammation	‐	‐	‐	‐
UTI during pregnancy (yes)	25	6.9	23	6.4
Premature prelabour rupture of membranes (yes)	7	1.9	5	1.4
Perinatal mortality (yes)	5	1.4	3	0.9
Offspring infection	‐	‐	‐	‐
Suspected early sepsis <72 h after birth	1	0.3	33	9.1
GA at delivery (weeks) median–IQR	39	38‐40	4^a^	1.1
Birth weight (grams) median–IQR	3395	3034‐3720	6^a^	1.7
Early neurodevelopmental morbidity	0	‐	0	‐
Gastrointestinal morbidity	‐	‐	‐	‐
NEC stage 2 (yes)	1	0.3	2	0.6
Respiratory morbidity	9	2.6	0	‐
*Pregnancy characteristics*				
Indicated preterm delivery^b^	7	2.0	0	‐
First‐trimester miscarriage <16 weeks	4	1.1	0	‐
Termination of pregnancy <24 weeks	1	0.3	0	‐
Stillbirth	1	0.3	0	‐
Term delivery	310	88.8	0	‐
Cervical length ≤25 mm	4	1.1	132^a^	37.8
Gestational diabetes	‐	‐	1	0.3
No	328	94.0	‐	‐
With diet	11	3.2	‐	‐
With medication	4	1.1	‐	‐
Hypertensive disorders	‐	‐	0	‐
None	310	88.8	‐	‐
Pregnancy‐induced hypertension	24	6.9	‐	‐
Preeclampsia	15	4.3	‐	‐
Place of delivery	‐	‐	4^a^	1.1
Home	38	10.9	‐	‐
Hospital, midwifery‐led care	49	14.0	‐	‐
Hospital, obstetrician‐led care	245	70.2	‐	‐
Hospital, referral center	13	3.7	‐	‐
Onset of labor	‐	‐	5^a^	1.4
Spontaneous contractions	188	53.9	‐	‐
Spontaneous rupture of membranes	68	19.5	‐	‐
Induction	77	22.1	‐	‐
Elective cesarean section	11	3.2	‐	‐
Mode of delivery	‐	‐	5^a^	1.4
Spontaneous	246	70.5	‐	‐
Ventouse extraction	50	14.3	‐	‐
Elective/planned cesarean section	14	4.0	‐	‐
Emergency cesarean section	34	9.7	‐	‐
Meconium stained fluid (yes)	52	14.9	13^a^	3.7
Small for gestational age (yes)^c^	41	12.2	1	0.3
Fetal sex (male)	189	54.2	4^a^	1.1
Admission to NICU (yes)^d^	8	2.3	0	‐

*Note*: This table includes data on all participants with complete records of the entire pregnancy.

Abbreviations: GA, gestational age; IQR, interquartile range; NICU, Neonatal Intensive Care Unit.

^a^ Including four miscarriages <16 weeks.

^b^ Due to maternal or fetal reasons.

^c^ For all pregnancies that ended ≥23 weeks of gestation (*n* = 337).

^d^ For all pregnancies that ended ≥24 weeks of gestation (*n* = 336).

The associations (OR and aOR) between risk factors and sPTB are presented in Tables 3 and S1. sPTB occurred more frequently among participants with the following risk factors (crude OR, Table 3): any ethnic minority (OR: 3.7, 95% CI: 1.4–9.6), low SES (OR: 3.7, 95% CI: 1.6–8.5), being single (OR: 4.5, 95% CI: 1.3–14.9), vaginal bleeding during the first or second trimester (OR: 3.9, 95% CI: 1.6–9.8) and one or more urinary tract infection(s) (UTIs) during pregnancy (OR: 4.7, 95% CI: 1.7–13.1).

**Table 3:** Odds ratios for several risk factors statistically significantly associated with spontaneous preterm birth

Risk factors	OR	95% CI	aOR	95% CI	aOR	95% CI
*Risk factors present at first prenatal visit*	‐	‐	Regular		Firth	
Ethnicity						
White‐European	Reference		‐	‐	‐	‐
Any ethnic minority	**3.7** *	**1.4–9.6**	2.4	0.8‐6.8	2.4	0.9–6.6
SES						
Middle/high	Reference		‐	‐	‐	‐
Low	**3.7** *	**1.6–8.5**	‐	‐	‐	‐
Marital status						
Married/living with partner	Reference		‐	‐	‐	‐
Single	**4.5** *	**1.3–14.9**	3.0	0.8–10.3	3.1	0.9–10.4
*Risk factors developed during pregnancy*						
Vaginal bleeding						
No	Reference		‐	‐	‐	‐
Vaginal bleeding during first and/or second trimester	**3.9** *	**1.6–9.8**	**3.6** *	**1.4–9.1**	**3.6** *	**1.4–9.0**
Urinary tract infections during pregnancy						
No	Reference		‐	‐	‐	‐
Yes	**4.7** *	**1.7–13.1**	**4.8** *	**1.7–13.9**	**4.9** *	**1.7–13.9**

*Note*: Outcome of univariate and multivariate logistic regression analyses.

Abbreviations: aOR, adjusted odds ratio; CI, confidence interval; OR, odds ratio; SES, socioeconomic status.

* *p* < 0.05.

Adjusting for SES somewhat attenuated most associations with sPTB: any ethnic minority (aOR: 2.4, 95% CI: 0.8–6.8), being single (aOR: 3.0, 95% CI: 0.8–10.3), and vaginal bleeding in the first or second trimester (aOR: 3.6, 95% CI: 1.4–9.1) on sPTB (Table 3). The effect sizes were similar to regular aORs using Firth's correction (Table 3). Risk factors with no statistically significant association with sPTB are presented in Table S1.

## 4 DISCUSSION

In this Dutch cohort, several established risk factors were confirmed for sPTB among nulliparous pregnant women deemed at low risk of sPTB; we found low SES, vaginal bleeding in the first or second trimester, and UTI during pregnancy increased the chance of sPTB.

Previous studies among nulliparous women identified several of the associated factors with sPTB found in our cohort, such as socioeconomic deprivation, ethnicity, marital status, vaginal bleeding during early pregnancy.^7^, ^10^, ^29^, ^30^, ^31^ A Danish study showed that vaginal bleeding during the first pregnancy also increased the risk of PTB in a second pregnancy, but this finding was not confirmed in a smaller study executed in the United States of America.^31^, ^32^ Some reported associations were absent in our study, including maternal age, BMI, maternal height, smoking during pregnancy, alcohol use, maternal medical history, ART, previous miscarriages or terminations of pregnancy, short cervical length.^7^, ^10^, ^30^, ^33^, ^34^ Unfortunately, our study was underpowered to detect these associations. This is most likely for the associations with small ORs found in other studies (e.g., maternal age and BMI). The lack of association between short cervical length and sPTB in our study is explained by the high rate of missing data for cervical length measurement.

Furthermore, the definition of nulliparity may have played a role in the absence of some associations in our study. In our study, pregnant women with a history of late second‐trimester miscarriage >16 weeks of gestation were not eligible, because this is an established risk factor for subsequent PTB.^14^, ^15^ The risk factor “any history of miscarriage” in our study comprises a first‐trimester miscarriage or an early second‐trimester miscarriage between 12 and 16 weeks of gestation. Most first trimester miscarriages are likely caused by chromosomal and other structural anomalies, whereas cervical insufficiency may play a more important role in second‐trimester pregnancy loss.^35^, ^36^, ^37^ This implies that previous first trimester miscarriages have a limited role in sPTB risk, in contrast to second‐trimester pregnancy loss.^14^, ^38^ Preventive measures should be investigated and made available to pregnant women with a history of pregnancy loss between 16 and 24 weeks of gestation.

The major strength of our cohort study is the inclusion of women from various ethnic backgrounds and SES. Both are important risk factors for sPTB and are also associated with other risk factors, for example, marital status, maternal height, and previous terminations of pregnancy. Moreover, we included exclusively women with pregnancies deemed to be healthy, who were not eligible for additional surveillance or preventive measures for sPTB. We found that a significant proportion of these women in fact developed complications including sPTB. Our extensive collection of additional data offers interesting possibilities for future research.

The foremost limitation of our study is the premature ending of the PROPELLOR study by the funding agent, which led to recruitment of just 9.1% of the sample size initially planned. Discontinuation of both randomized and nonrandomized studies due to recruitment failure affects up to 10% of studies in medical research.^39^, ^40^, ^41^ The sense of urgency for midwives to investigate sPTB may have been limited, due to the expected lower incidence of PTB in their healthy population. This has been seen in other studies of low incidence conditions, although a range of other factors could have contributed to slow recruitment, including the burden of a scientific study without adequate financial compensation and lack of sufficient timely public patient involvement.^42^ To reduce research waste, we are actively seeking collaboration to utilize the substantial numbers of bio‐samples and additional data collected in our study (Appendix S3).

Despite its modest size, we did collect a cohort large enough to detect risk factors with an effect size ≥3. This is relevant for several risk factors investigated, for example, vaginal bleeding in the first or second trimester, since previous studies found similar effect sizes.^9^ It is also relevant to explore risk factors with smaller effect sizes in studies that also include high‐risk nulliparous and multiparous pregnant women. These risk factors might have a larger effect in a cohort of healthy nulliparous women only, because of the lack of obstetric or medical history for risk stratification.

## 5 CONCLUSION

This Dutch cohort study confirms that a substantial proportion of nulliparous pregnant women deemed at low risk of sPTB in fact delivered preterm, and we show that well‐known risk factors associated with sPTB apply to low‐risk nulliparous women. We found that low SES, vaginal bleeding in the first or second trimester, and UTIs during pregnancy increased the chance of sPTB, but early miscarriage <16 weeks of gestation did not.

## AUTHOR CONTRIBUTIONS

*Conceptualization*: Petra J. Hajenius, Marjolein Kok, Joris A. M. van der Post, Rebecca C. Painter. *Funding acquisition*: Petra J. Hajenius, Marjolein Kok, Rebecca C. Painter. *Formal analysis*: Heleen J. Schuster, Monique D. M. van Beukering, Rik van Eekelen, Marit Schonewille. *Investigation*: Heleen J. Schuster, Myrthe J. C. S. Peelen, Henna Playfair, Marit Schonewille. *Project administration*: Myrthe J. C. S. Peelen, Petra J. Hajenius, Marjolein Kok. *Supervision*: Petra J. Hajenius, Marjolein Kok, Rebecca C. Painter. *Writing—original draft preparation*: Heleen J. Schuster, Marit Schonewille. *Writing—review & editing*: Myrthe J. C. S. Peelen, Petra J. Hajenius, Monique D. M. van Beukering, Rik van Eekelen, Henna Playfair, Joris A. M. van der Post, Marjolein Kok, Rebecca C. Painter. All authors have read and approved the final version of the manuscript. Heleen J. Schuster had full access to all of the data in this study and takes complete responsibility for the integrity of the data and the accuracy of the data analysis.

## CONFLICTS OF INTEREST

The authors declare no conflicts of interest.

## ETHICS STATEMENT

All methods were carried out in accordance with relevant guidelines and regulations. All participants provided written informed consent. The study received ethical clearance through Medical Ethics Committee of the Academic Medical Center in Amsterdam, the Netherlands (registration number NL43414.018.13).

## Supporting information

Supplementary information.

Supplementary information.

Supplementary information.

Supplementary information.

Supplementary information.

## Data Availability

We encourage future collaboration with other research groups. Detailed information on the pregnancy is available upon request, together with data collected from the questionnaires (see Appendix S1) and cervical length measurements. Blood samples are aliquoted in several serum, plasma, and buffy coat samples and available for analysis. The study protocol and informed consent form are available on request. Data can be made available in accordance with the consent provided by participants and legal regulations, immediately following publication until 20 years after start of the study for sPTB related research. All biomaterial is available for sPTB biomarker research immediately following publication until 20 years after start of the study. A subset of participants consented to an additional biobank, allowing biomaterial research with other purposes. For sharing of data or biomaterial a legal agreement according to law and regulations must be signed. The data that support the findings of this study are available upon reasonable request. Proposals for future research or data availability requests should be directed to r.c.painter@amsterdamumc.nl.
