# Supplementary material for: Risk factors for spontaneous preterm birth among healthy nulliparous pregnant women in the Netherlands, a prospective cohort study
Source: Health Sci Rep. 2022 May 24;5(3):e585. doi: 10.1002/hsr2.585 (PMC9127744; doi:10.1002/hsr2.585)
Supplement: Supplementary file 2 — Supplementary information. [file HSR2-5-e585-s002.pdf]

PROPELLOR - Prevention of premature birth

Questionnaire 1

Completed at inclusion

1. General details

1.1 In which country were **you** born and in which country was your **husband/partner** born?

**I**	**Husband/partner**
☐ The Netherlands	☐ The Netherlands
☐ Suriname	☐ Suriname
☐ The Antilles/Aruba	☐ The Antilles/Aruba
☐ Morocco	☐ Morocco
☐ Turkey	☐ Turkey
☐ Indonesia	☐ Indonesia
☐ India	☐ India
☐ China	☐ China
☐ Ghana	☐ Ghana
☐ Nigeria	☐ Nigeria
☐ Cape Verde	☐ Cape Verde
☐ Germany	☐ Germany
☐ Great Britain	☐ Great Britain
☐ Belgium	☐ Belgium
☐ Other, namely	☐ Other, namely ....

1.2 In which country were your parents born?

**Mother**	**Father**
☐ The Netherlands	☐The Netherlands
☐ Suriname	☐ Suriname
☐ The Antilles/Aruba	☐ The Antilles/Aruba
☐ Morocco	☐ Morocco
☐ Turkey	☐ Turkey
☐ Indonesia	☐ Indonesia
☐ India	☐ India
☐ China	☐ China
☐ Ghana	☐ Ghana
☐ Nigeria	☐ Nigeria
☐ Cape Verde	☐ Cape Verde
☐ Germany	☐ Germany
☐ Great Britain	☐ Great Britain
☐ Belgium	☐ Belgium
☐ Other, namely	☐ Other, namely ....
☐ I don't know	☐ I don't know

1.3 In which country were the parents of <u>your husband/partner</u> born?

Mother
- ☐ The Netherlands
- ☐ Suriname
- ☐ The Antilles/Aruba
- ☐ Morocco
- ☐ Turkey
- ☐ Indonesia
- ☐ India
- ☐ China
- ☐ Ghana
- ☐ Nigeria
- ☐ Cape Verde
- ☐ Germany
- ☐ Great Britain
- ☐ Belgium
- ☐ Other, namely ……………..
- ☐ I don't know

Father
- ☐The Netherlands
- ☐ Suriname
- ☐ The Antilles/Aruba
- ☐ Morocco
- ☐ Turkey
- ☐ Indonesia
- ☐ India
- ☐ China
- ☐ Ghana
- ☐ Nigeria
- ☐ Cape Verde
- ☐ Germany
- ☐ Great Britain
- ☐ Belgium
- ☐ Other, namely ……………
- ☐ I don't know

1.4 To which ethnicity do you subscribe yourself?

☐	Caucasian	(Dutch, white European, Turkey excluded)
☐	African	(African, Surinamese/Antillean of negroid origin)
☐	Hindustani	(Hindustani, Pakistani, Indian, Surinamese/Antillean of hindustani origin)
☐	Moroccan	(Moroccan, Algerian, North African)
☐	Turkish	(Turkish, Kurdish)
☐	Middle East	(Iranian, Iraqi)
☐	Asian	(Chinese, Japanese, Indonesian, Surinamese/Antillean of Javanese or Chinese origin, South East Asia)
☐	Other Western	(North American, Australian)
☐	Other non-Western	(South and Central American)
☐	Mixed	(mixed origin)

1.5 What is your civil status?

- ☐ Married
- ☐ Cohabiting
- ☐ Steady relationship, not cohabiting
- ☐ Single

2. Education

2.1 What is the highest level of education you have completed?

☐ I have not been able to complete a level of education.
☐ Primary school
☐ Secondary education
  ☐ Preparatory vocational education/lower secondary vocational education/advanced vocational track of the preparatory secondary vocational education (in the Dutch education system referred to as VBO/LBO/VMBO kaderberoepsgerichte leerweg)
  ☐ Junior general secondary education/combined track (practice and theory) or theoretical track of the preparatory secondary vocational education (in the Dutch education system referred to as MAVO/VMBO)
  ☐ Senior general secondary education (in the Dutch education system referred to as HAVO)
  ☐ Pre-university education (in the Dutch education system referred to as VWO)
☐ Senior secondary vocational education (in the Dutch education system referred to as MBO)
☐ Higher professional education (in the Dutch education system referred to as HBO)
☐ University education or higher academic education

3. Current Pregnancy

3.1. How many months ago was your last pregnancy (for example a miscarriage, abortion or ectopic pregnancy)?

☐ Not applicable, this is my first pregnancy
☐ < 6 months ago
☐ ≥ 6 - 12 months ago
☐ ≥ 12 - 18 months ago
☐ ≥ 18 - 24 months ago
☐ ≥ 24 months ago

3.2 How many months did it take before you were pregnant, after you had decided you wanted to become pregnant?

………… months
Or ☐ this pregnancy is unintentional

3.3 Did this pregnancy start as a 'vanishing twin' (in the first instance a twin pregnancy)?

☐ No
☐ Yes

3.4 Have you experienced vaginal bleeding during your current pregnancy?

☐ No
☐ Yes, namely ……… consecutive days

3.5 How did your current pregnancy come about?

☐ Naturally (spontaneously, without medical treatment)
→ ***continue with question 4.1 on page 11***

☐ With a fertility treatment: **(more than one answer possible)**
- ☐ Ovulation induction with clomid tablets (hormones for inducing ovulation)
- ☐ Ovulation induction with gonadotropin injections (for example puregon, menopur, gonal f, fostimon)
- ☐ Ovulation induction with injections and a hormone pump (GnrH)
- ☐ Ovulation induction after an exploratory operation with electrocoagulation of the ovaries (LEO)
- ☐ ICI (the semen has been inserted into the vagina at the cervix)
- ☐ IUI (the semen has been inserted into the uterus) without hormonal stimulation
- ☐ IUI **with** stimulation with clomid tablets
- ☐ IUI **with** gonadotropin injections (puregon, menopur, gonal f, fostimon)
- ☐ IVF or ICSI

3.6 In which hospital or clinic did you undergo the fertility treatment?

Hospital:………………………………………………….. in (location):………………………………………….

3.7 Which egg cell and which sperm have been combined to make you pregnant?
In **both** rows, tick the box with the answer that is most appropriate for your situation

☐ Own egg cell	☐ Sperm of partner ('fresh')
☐ Own frozen egg cell (vitrified)	☐ Sperm of partner obtained through PESA/MESA (operation on the epididymis)
☐ Donated egg cell	☐ Sperm of partner obtained through TESE (operation on the testicle)
	☐ Sperm of a private donor
	☐ Sperm of a sperm bank donor

3.8 Have you become pregnant after an IVF or ICSI treatment?

☐ No → ***continue with question 4.1*** ☐ IVF ☐ ICSI

3.9 Have you become pregnant after the placing of an embryo following the follicular puncture (fresh placement)?

☐ Yes, I have become pregnant after the fresh placement
☐ No, I have become pregnant after the placement of a thawed embryo ("cryo-embryo")

3.10 How many days old was the embryo upon placement?
☐ Day 3 fresh embryo
☐ Day 4 thawed day 3 embryo
☐ Day 5 fresh embryo blastocyst stage
☐ Day 6 thawed blastocyst
☐ I don't know

3.11 Was the IVF or ICSI treatment combined with Pre-Implantation Genetic Diagnostics (PGD; with this diagnostics, a cell of the embryo is examined for hereditary abnormalities)?

☐ No
☐ Yes

4. General health / lifestyle

4.1 How tall are you? (cm) ☐☐☐

4.2 How much did you weigh just before you became pregnant? (kg) ☐☐☐

4.3 Do you take medication?

- ☐ No → ***continue with question 4.5***
- ☐ Yes, medication prescribed by my general practitioner / doctor in the hospital
- ☐ Yes, over-the-counter medication available to me at a pharmacy or chemist (for example paracetamol, cough syrups, salves, vitamins, homeopathic remedies)

4.4 Which medication do you take? **(more than one answer possible)**

- ☐ Antibiotics (for example penicillin)
- ☐ Painkillers (for example paracetamol)
- ☐ Anti-inflammatory agents (for example ibuprofen, advil)
- ☐ Medication for vomiting and nausea (for example emesafene, zantac)
- ☐ Sleep-inducing drugs and sedatives (for example valium)
- ☐ Antidepressants (for example prozac, seroxat)
- ☐ Thyroid hormones (for example thyrax)
- ☐ Homeopathic remedies
- ☐ Vitamins (Folic acid, vitamin B6, vitamin D)
- ☐ I don't know which medication I am taking
- ☐ Other, namely ………….

4.5 Do you smoke?

- ☐ No, I have never smoked
- ☐ No, I quit smoking when I realized I was pregnant
- ☐ No, I quite before

  When did you quit? (calendar year) ☐☐☐☐
- ☐ Yes
  - ☐ 0-9 cigarettes a day
  - ☐ 10-19 cigarettes a day
  - ☐ > 20 cigarettes a day

4.6 How often does someone smoke in your home (including smoking under the cooker hood)?

☐ Never
☐ Less than monthly
☐ Monthly
☐ Weekly
☐ Daily

4.7 In the past thirty days, has anybody smoked inside where you work?

☐ No
☐ Yes

4.8 Do you drink alcohol?

☐ No
☐ No, I quit drinking alcohol when I realized I was pregnant
☐ Yes, sometimes, < 1 glass a day
☐ Yes, 1-5 glasses a day
☐ > 5 glasses a day

4.9 Do you take drugs?

☐ No, never → ***continue with question 5.1***
☐ No, I quit before → ***continue with question 5.1***
☐ Yes, recently during my pregnancy

4.10 If you answered yes, which drugs did you use?
Per used drug, tick ☒ the right box for the level of use:

	Very sporadically	Monthly	Weekly	(almost) Daily
1. Cannabis/hash/marijuana/weed				
2. Heroin				
3. Methadon				
4. Cocaine				
5. Crack, base coke				
6. XTC (ecstasy)				
7. Speed (amphetamine)				
8. Magic mushrooms, herbal drugs				
9. LSD or other hallucinogens				
10. Methamphetamine (meth)				
11. Other				

5. Diet

Tick the box with the answer that most suits your situation from the start of your pregnancy up until now.

5.1 How long have you felt nauseated on an average day?

☐ Not at all ☐ ≤ 1 hour ☐ 2-3 hours ☐ 4-6 hours ☐ > 6 hours

5.2 How often have you vomited or been sick on an average day?

☐ Not at all ☐ 1-2 times ☐ 3-4 times ☐ 5-6 times ☐ ≥ 7 times

5.3 How often on an average day have you had to retch or did you feel queasy without actually vomiting?

☐ Not at all ☐ 1-2 times ☐ 3-4 times ☐ 5-6 times ☐ ≥ 7 times

6. Bowel movement pattern

6.1 On average, how often do you have bowel movement per week: …… times

6.2 Do you sometimes have to push very hard in order to be able to poop?
☐ No ☐ Yes

6.3 Are you suffering from hard stool?
☐ No ☐ Yes

6.4 Are you experiencing a feeling of blockage?
☐ No ☐ Yes

6.5 Do you sometimes have the feeling that stool is left behind in your rectum or intestines?

☐ No ☐ Yes

6.6 Do you sometimes use your fingers or other objects in order to be able to poop? ☐ No ☐ Yes

If you answered the questions above (6.2 up to and including 6.6) with 'no' then please

→ ***continue with question 7.1***

If you answered at least one of the questions above (6.2 up to and including 6.6) with 'yes':

6.7 When did the symptoms first arise?
☐ Before the pregnancy
☐ During the pregnancy

7. Working conditions and workload

A. Work (outdoors):

7.1 Do you currently perform paid work?

- ☐ No → ***continue with section B on page 23***
- ☐ Yes → ***continue with question 7.2***

7.2 Have you had your current job during the entirety of this pregnancy?

- ☐ No, I've had this job since I was …… weeks pregnant
- ☐ Yes

7.3 In which sector do you currently work?

- ☐ Government
- ☐ Health care
- ☐ Education
- ☐ Welfare and child care
- ☐ Business services
- ☐ Retail (shops)
- ☐ Culture, recreation
- ☐ Hotel and catering industry
- ☐ Industry/construction
- ☐ Cleaning
- ☐ Other, namely……………………………………………….

7.4 In which position do you currently work?

- ☐ Manager
- ☐ (Para)medic
- ☐ Caring and nursing position
- ☐ Primary/secondary/adult education teacher
- ☐ Child day care centre employee
- ☐ Administrative assistant
- ☐ Counter/reception work
- ☐ Shop assistant or other salesperson
- ☐ Social/cultural worker
- ☐ Accountant or other financial employee
- ☐ Serving employee
- ☐ Domestic services employee
- ☐ Domestic services employee in hotel (cleaning services)
- ☐ Production assistant or factory employee
- ☐ Flower auction employee
- ☐ Other, namely ……………………………

7.5 How many employees does the company where you work have?

- ☐ 1-10
- ☐ 11-50
- ☐ 51-100
- ☐ More than 100

7.6a. How many hours per week do you currently work on average? ………… hours a week

7.6b. How many days per week do you work on average? ………… days a week

7.7 Do you work in irregular shifts?

- ☐ No
- ☐ Yes

  How many of your working hours do you on average spend on these shifts **a week**?

  - ■ ………. hours a week in day shifts
  - ■ ………. hours a week in evening shifts (until 23:00)
  - ■ ………. hours a week in night shifts

7.8a. During your work, how many hours a day do you have to:

- ☐ Walk ...... hours a day
- ☐ Stand ……hours a day
- ☐ Sit ......hours a day

7.8b. If you have to sit, do you have the opportunity to sometimes get up and stand or walk for a while?

- ☐ Always
- ☐ Sometimes
- ☐ Rarely
- ☐ Never

7.8c. If you have to walk or stand, do you have the opportunity to sometimes sit down for a while:

- ☐ Always
- ☐ Sometimes
- ☐ Rarely
- ☐ Never

7.9a. Do you have to lift and/or carry things during your work?

- ☐ No → ***continue with question 7.10***
- ☐ Yes → ***continue with question 7.9b***

7.9b. During my work I lift or carry:

- ☐ People
- ☐ Animals
- ☐ Loads

7.9c. In the table below you can fill in how much weight you lift and how often this occurs. More than one answer is possible. If you are lifting loads or people heavier than 20kg, please fill in how much kg they weigh on average.

	Less than 5 kg	5-10 kg	10-20 kg	More than 20 kg
1-10 times a day (once an hour)	☐	☐	☐	☐ ....kg
10-25 times a day (2 or 3 times an hour)	☐	☐	☐	☐ ....kg
25-50 times a day (4 to 6 times an hour)	☐	☐	☐	☐ ....kg
50-100 times a day (7 to 12 times an hour)	☐	☐	☐	☐ ....kg
More than 100 times a day (13 times an hour)	☐	☐	☐	☐ .... kg

7.10 For the next couple of questions, please indicate every time <u>how often</u> certain things occur at the moment. You can choose between the following answers:
never, sometimes, often, regularly.
Try not to skip any questions.

		never	sometimes	regularly	often
a.	Do you have to bend over during work?	☐	☐	☐	☐
b.	Do you have to squat during work?	☐	☐	☐	☐
c.	Do you have to reach out high with your arms during work?	☐	☐	☐	☐
d.	Do you have to make the same repetitive motion a lot during your work?	☐	☐	☐	☐
e.	Do you think your work is very physically demanding?	☐	☐	☐	☐
f.	Does your work require physical strength?	☐	☐	☐	☐
g.	Do you work in uncomfortable or strenuous postures?	☐	☐	☐	☐
h.	Do you work at a strenuous machine or assembly line?	☐	☐	☐	☐

7.11 During your work, are you often exposed to one of the factors below?

		never	sometimes	regularly	often
a.	High temperatures/heat	☐	☐	☐	☐
b.	Low temperatures/coldness	☐	☐	☐	☐
c.	Noise	☐	☐	☐	☐
d.	Body vibrations	☐	☐	☐	☐
e.	Overpressure	☐	☐	☐	☐

7.12 For the next couple of questions, please indicate every time <u>how often</u> certain things occur at this moment. You can choose between the following answers:
never, sometimes, often, always. Try not to skip any questions.

		never	sometimes	often	always
a.	Do you have problems with the pace of work?	☐	☐	☐	☐
b.	Do you have problems with the pressure of work?	☐	☐	☐	☐
c.	Would you like to take things a little easier in your work?	☐	☐	☐	☐
d.	Are you free in the performance of your tasks?	☐	☐	☐	☐
e.	Do you have any influence on the pace of work?	☐	☐	☐	☐
f.	Can you plan your work yourself?	☐	☐	☐	☐
g.	Do you experience sufficient support from your manager?	☐	☐	☐	☐
h.	Do you experience sufficient support from your colleagues?	☐	☐	☐	☐
i.	Do you enjoy your work?	☐	☐	☐	☐
j.	Are you satisfied with your work?	☐	☐	☐	☐

7.13 Is there a chance for you to contact an infectious disease at your work?

- ☐ No
- ☐ Yes; I regularly work with: **(more than one answer possible):**
  - ☐ Ill and/or small children
  - ☐ Ill adults
  - ☐ Animals
  - ☐ Nature, in forests or public gardens
  - ☐ Raw meat
  - ☐ Waste or waste water
  - ☐ Blood or other bodily fluids (also via waste)
  - ☐ Stools (also via waste)

7.14 Do you come into contact with chemical substances during your work?

- ☐ No
- ☐ Yes, with: **(more than one answer possible)**
  - ☐ Cleaning supplies
  - ☐ Solvents (for example in paint, lacquer, glue, detergents and ink)
  - ☐ Anaesthetic gasses
  - ☐ Cancer-inhibitory medication (medication used during chemotherapy)
  - ☐ Pesticides
  - ☐ Heavy metals and/or metallic compounds (for example cadmium, mercury, lead, mangenese and chromium)
  - ☐ Other, namely……………………………………………………………
- ☐ I don’t know

7.15 On average, in which fashion do you currently travel to work?

☐ Walking
☐ By bicycle/scooter
☐ Public transport
☐ Car

7.16 How great is the distance (the total of the trip there and back) that you have to travel for this? **(tick the box that is most appropriate for your situation and fill in the number of (kilo)metres)**

☐ ……………… metre ☐ ……………… kilometer

7.17 My daily travel time to and from work (total of the trip there and back) is on average: **(tick the box that is most appropriate for your situation and fill in the number of minutes or hours)**

☐ ……………… minutes ☐ ……………… hours

7.18 Have any adjustments been made to your work because you had complaints or were suffering from a disease/disorder before your current pregnancy?

☐ No
☐ Yes, due to …………………………………………………………………………….

The following adjustments have been made: **(more than one answer possible)**

☐ Less physically taxing work
☐ Less standing or walking
☐ Lifting or carrying less or less often
☐ Slower work pace
☐ Less work
☐ Other working hours
☐ Less hours a day
☐ No more night shifts
☐ More opportunity to plan the work at your own discretion
☐ More working from home
☐ Other adjustments, namely ……………………..…………………………………………

7.19 Have you had an increase in complaints that limit you in your work because of your pregnancy?

☐ No

☐ Yes, namely…… **(more than one answer possible)**

- ☐ Complaints of fatigue
- ☐ Headaches
- ☐ Complaints of pain in the back, pelvis and/or legs
- ☐ Nausea/vomiting
- ☐ Stomach aches
- ☐ Other complaints, namely………………………………………………………………………

7.20 Have adaptions been made to your work because you are currently pregnant?

☐ No → ***continue with section B on page 23***

☐ Yes, namely…… **(more than one answer possible)**

- ☐ Less physically taxing work
- ☐ Less standing or walking
- ☐ Lifting or carrying less or less often
- ☐ Slower work pace
- ☐ Less work
- ☐ Other working hours
- ☐ Less hours a day
- ☐ No more night shifts
- ☐ More opportunity to plan the work at your own discretion
- ☐ More working from home
- ☐ Other adjustments, namely ……………………………………………………………………

These adjustments have been in effect since …………. weeks of pregnancy

7.21 If adjustments have been made to your work, did you feel the need for these adjustments?

☐ No

☐ Yes

7.22 Who advised you to adjust your work because of your pregnancy?
(more than one answer possible)

☐ Company doctor

☐ General practitioner

☐ Manager

☐ Staff advisor

☐ Yourself (at your own initiative)

☐ Other

B. Spare time

7.23 Do you currently participate in a sport while pregnant?

☐ No, and I don't normally participate in sports either
☐ No, I stopped participating in sports as soon as I realised I was pregnant
☐ Yes, namely: (fill in which sport)…………………………………………………………………
  ☐ Less than two hours a week
  ☐ More than two hours a week

7.24 Do you have a hobby on which you spend more than five hours a week?

☐ No
☐ Yes, namely………………………………………………………………………………………………………

C. Domestic situation

7.25 Who is currently doing the housekeeping?

☐ You are largely doing it yourself
☐ You are doing it together with your partner/someone else
☐ Your partner/someone else is doing the most of it

7.26 Do you have a household help?

☐ No
☐ Yes, for …………. hours a week (on average)

7.27 Are you caring for any other children living at home (for example your partner's or a relative's children)?

☐ No
☐ Yes, …………. (please fill in the number of children)

8. Medical Information

8.1 Have you ever had surgery on the cervix?

☐ No
☐ Yes, a curettage
☐ Yes, a cryocoagulation (freezing) after an abnormal cervical smear
☐ Yes, a large loop excision of the transformation zone (LLETZ) (electrically burning away a piece of the cervix) after an abnormal cervical smear
☐ Yes, a cervical conisation (removing a cone-shaped piece of the cervix) after an abnormal cervical smear
☐ Yes, but I don't know which type of surgery

8.2 Have you ever had an operation which was conducted in the abdomen (for example an operation on the cecum or intestines, the gall bladder, an ovarian cyst or an ectopic pregnancy)?

☐ No
☐ Yes, by means of an exploratory surgery due to ……………………………………………
☐ Yes, by means of an open abdominal surgery due to ……………………………………

8.3 Have you ever had a bladder infection? **(more than one answer possible):**

☐ No, never
☐ Yes, but less than 3 times a year **before** my pregnancy
☐ Yes, 3 times or more a year **before** my pregnancy
☐ Yes, during this pregnancy

8.4 Have you ever suffered from a venereal disease before the pregnancy? **(more than one answer possible)**

☐ No
☐ Yes, namely:
  ☐ Chlamydia
  ☐ Gonorrhoea
  ☐ Syphilis (lues)
  ☐ Herpes
  ☐ Vaginal warts

8.5 Have you ever suffered from a vaginal fungal infection (=Candidiasis) before the pregnancy?

☐ No
☐ Yes

8.6a Have your undergone or are you undergoing treatment at a psychologist, psychiatrist or your GP in connection with a psychological problem? (for example depression, an anxiety disorder, psychosis, burnout)

☐ No → ***continue with question 8.7***
☐ Yes, in the past
☐ Yes, currently

8.6b Did you or have you received medication for this?

☐ No
☐ Yes

8.7 Is premature birth (< 37 weeks of pregnancy) prevalent in your or your partner's family? **(more than one answer possible):**

☐ No
☐ Yes
  ☐ own mother
  ☐ own sister(s)
  ☐ mother of partner
  ☐ sister(s) of partner

8.8 Were you yourself born prematurely?

☐ No → ***continue with question 9.1***
☐ Yes
  At how many weeks were you born? ………… weeks
  (normal pregnancy is 40 weeks)
  ☐ I don't know
☐ I don’t know → ***continue with question 9.1***

8.9 Was your own birth a 'spontaneous' premature birth?

☐ No, my mother was induced (the labour is stimulated)
☐ Yes
☐ I don’t know

9. Relationship

9.1 Is there a partner from a previous relationship who causes you to feel unsafe?
- ☐ No
- ☐ Yes
- ☐ Not applicable, I do not have a previous partner

9.2 Do you feel safe in your current relationship?
- ☐ No
- ☐ Yes
- ☐ Not applicable; I am not in a steady relationship at the moment
  → ***continue with question 9.12***

9.3 How would you describe your current relationship in general?
- ☐ No tension/stress
- ☐ Some tension/stress
- ☐ A lot of tension/stress

9.4 Does resolving arguments cost you and your partner:
- ☐ No effort
- ☐ Some effort
- ☐ A lot of effort

9.5 Do arguments within your relationship cause you to feel down or bad about yourself?
- ☐ Never
- ☐ Sometimes
- ☐ Often

9.6 Do arguments result in hitting, kicking or pushing?
- ☐ Never
- ☐ Sometimes
- ☐ Often

9.7 Do you ever feel frightened by what your partner says or does?
- ☐ Never
- ☐ Sometimes
- ☐ Often

9.8 Has your partner ever physically abused you (for example hit you, kicked you or punched you)?

☐ Never
☐ Sometimes
☐ Often

9.9 Has your partner ever emotionally abused you (for example repeatedly called you names, tormented you, manipulated you, humiliated you, insulted you, locked you up or ignored you)?

☐ Never
☐ Sometimes
☐ Often

9.10 Has your partner ever made you perform sexual acts (for example kissing, touching or undressing) that you did not want to do or which made you feel uncomfortable?

☐ Never
☐ Sometimes
☐ Often

9.11 Has your partner ever sexually abused you (assaulted or raped you: penetration of anus, vagina or mouth with finger, object or genitals against your will)?

☐ Never
☐ Sometimes
☐ Often

9.12 Have you been hit, kicked, punched or otherwise been hurt by someone in the last year?

☐ No
☐ Yes
If yes, by whom?
☐ A person in a current intimate relationship
☐ A person in a previous intimate relationship
☐ A person with whom I do not have or have had an intimate relationship

The following questions concern relationships during **<u>your entire life.</u>**

9.13 Have arguments within a relationship ever lead to hitting, kicking or pushing?

- ☐ Never
- ☐ Sometimes
- ☐ Often

9.14 Have you ever been physically abused (for example hit, kicked or punched) by a previous partner, a family member or an acquaintance?

- ☐ Never
- ☐ Sometimes
- ☐ Often

9.15 Have you ever been emotionally abused by a partner/family member or acquaintance (has he/she, for example, repeatedly called you names, tormented you, manipulated you, humiliated you, insulted you, locked you up or ignored you)?

- ☐ Never
- ☐ Sometimes
- ☐ Often

9.16 Has a partner/family member or acquaintance ever made you perform sexual acts (for example kissing, touching or undressing) that you did not want to do or which made you feel uncomfortable?

- ☐ Never
- ☐ Sometimes
- ☐ Often

9.17 Have you ever been sexually abused by a partner/family member or acquaintance?

- ☐ Never
- ☐ Sometimes
- ☐ Often

End of the questionnaire. Thank you for completing the form!

Space for remarks:

PROPELLOR - Prevention of premature birth

Questionnaire 2

Completed between 20 to 24 weeks of gestation

Questionnaire "Working conditions and workload"

Please note: this questionnaire is about the past 10 weeks (since you filled in part 1 of the questionnaire)!

A. Work (outdoors):

1. What is your current work status?

☐ I am still working
☐ I am working part-time due to complaints/illness
☐ I am no longer working due to complaints/illness
☐ I am on pregnancy leave
☐ I do not have a paid job → ***Please continue with part B***

2. Have you received any information when you informed your employer of your pregnancy?

☐ No
☐ No, I am an independent entrepreneur
☐ Yes, about **(more than one answer possible)**
☐ Pregnancy and maternity leave
☐ Combination breastfeeding/expressing and work
☐ extra rest periods
☐ working conditions and any possible adjustments to the work

3. Have adaptions been made to your work <u>because you are currently pregnant</u>?

☐ No → ***continue with question 6***
☐ Yes, namely…… ***(more than one answer possible)***
☐ Less physically taxing work
☐ Less standing or walking
☐ Lifting or carrying less or less often
☐ Slower work pace
☐ Less work
☐ Other working hours
☐ Less hours a day
☐ No more night shifts
☐ More opportunity to plan the work at your own discretion
☐ More working from home
☐ Other adjustments, namely ……………………………………………………………………

These adjustments have been in effect since …………. weeks of pregnancy

4. If adjustments have been made to your work, did you feel the need for these adjustments?

☐ No
☐ Yes

5. Who advised you to adjust your work because of your pregnancy?
(more than one answer possible)

- ☐ Obstetrician
- ☐ Gynaecologist
- ☐ Company doctor
- ☐ General practitioner
- ☐ Manager
- ☐ Staff advisor
- ☐ Yourself (at your own initiative)
- ☐ Other, namely .....

6. Have you had an increase in complaints that limit you in your work <u>because of your pregnancy</u>?

- ☐ No
- ☐ Yes, namely…… ***(more than one answer possible)***
  - ☐ Complaints of fatigue
  - ☐ Headaches
  - ☐ Complaints of pain in the back, pelvis and/or legs
  - ☐ Nausea/vomiting
  - ☐ Stomach aches
  - ☐ Contractions
  - ☐ Other complaints, namely…………………………………………………………………………

If you are currently no longer working → **Please continue with part B and then C**

7.a. How many hours per week do you currently work on average? ……… hours a week
7.b. How many days per week do you work on average? ……… days a week

8. Do you work in irregular shifts?

- ☐ No
- ☐ Yes

  How many of your working hours do you on average spend on these shifts <u>**a week**</u>?
  - ■ ………. hours a week in day shifts
  - ■ ………. hours a week in evening shifts (until 23:00)
  - ■ ………. hours a week in night shifts

9a. During your work, how many hours a day do you have to:

- ☐ Walk ...... hours a day
- ☐ Stand …… hours a day
- ☐ Sit ...... hours a day

9b. If you have to sit, do you have the opportunity to sometimes get up and stand or walk for a while?

- ☐ Always
- ☐ Sometimes
- ☐ Rarely
- ☐ Never

9c. If you have to walk or stand, do you have the opportunity to sometimes sit down for a while:

- ☐ Always
- ☐ Sometimes
- ☐ Rarely
- ☐ Never

10a. Do you have to lift and/or carry things during your work?

- ☐ No → ***continue with question 11***
- ☐ Yes → ***continue with question 10b***

10b. During my work I lift or carry:

- ☐ People
- ☐ Animals
- ☐ Loads

10c. In the table below you can fill in how much weight you lift and how often this occurs. More than one answer is possible. If you are lifting loads or people heavier than 20kg, please fill in how much kg they weigh on average.

	Less than 5 kg	5-10 kg	10-20 kg	More than 20 kg
1-10 times a day (once an hour)	☐	☐	☐	☐ ....kg
10-25 times a day (2 or 3 times an hour)	☐	☐	☐	☐ ....kg
25-50 times a day (4 to 6 times an hour)	☐	☐	☐	☐ ....kg
50-100 times a day (7 to 12 times an hour)	☐	☐	☐	☐ ....kg
More than 100 times a day (13 times an hour)	☐	☐	☐	☐ .... kg

11. For the next couple of questions, please indicate every time <u>how often</u> certain things occur at the moment. You can choose between the following answers:
never, sometimes, often, regularly.
Try not to skip any questions.

		never	sometimes	regularly	often
a.	Do you have to bend over during work?	☐	☐	☐	☐
b.	Do you have to squat during work?	☐	☐	☐	☐
c.	Do you have to reach out high with your arms during work?	☐	☐	☐	☐
d.	Do you have to make the same repetitive motion a lot during your work?	☐	☐	☐	☐
e.	Do you think your work is very physically demanding?	☐	☐	☐	☐
f.	Does your work require physical strength?	☐	☐	☐	☐
g.	Do you work in uncomfortable or strenuous postures?	☐	☐	☐	☐
h.	Do you work at a strenuous machine or assembly line?	☐	☐	☐	☐

12. During your work, are you often exposed to one of the factors below?

		never	sometimes	regularly	often
a.	High temperatures/heat	☐	☐	☐	☐
b.	Low temperatures/coldness	☐	☐	☐	☐
c.	Noise	☐	☐	☐	☐
d.	Body vibrations	☐	☐	☐	☐
e.	Overpressure	☐	☐	☐	☐

13. For the next couple of questions, please indicate every time <u>how often</u> certain things occur at this moment. You can choose between the following answers:
never, sometimes, often, always. Try not to skip any questions.

		never	sometimes	often	always
a.	Do you have problems with the pace of work?	☐	☐	☐	☐
b.	Do you have problems with the pressure of work?	☐	☐	☐	☐
c.	Would you like to take things a little easier in your work?	☐	☐	☐	☐
d.	Are you free in the performance of your tasks?	☐	☐	☐	☐
e.	Do you have any influence on the pace of work?	☐	☐	☐	☐
f.	Can you plan your work yourself?	☐	☐	☐	☐
g.	Do you experience sufficient support from your manager?	☐	☐	☐	☐
h.	Do you experience sufficient support from your colleagues?	☐	☐	☐	☐
i.	Do you enjoy your work?	☐	☐	☐	☐
j.	Are you satisfied with your work?	☐	☐	☐	☐

14. Is there a chance for you to contact an infectious disease at your work?

☐ No
☐ Yes; I regularly work with: ***(more than one answer possible):***
  ☐ Ill and/or small children
  ☐ Ill adults
  ☐ Animals
  ☐ Nature, in forests or public gardens
  ☐ Raw meat
  ☐ Waste or waste water
  ☐ Blood or other bodily fluids (also via waste)
  ☐ Stools (also via waste)

15. Do you come into contact with chemical substances during your work?

☐ No
☐ Yes, with: ***(more than one answer possible)***
  ☐ Cleaning supplies
  ☐ Solvents (for example in paint, lacquer, glue, detergents and ink)
  ☐ Anaesthetic gasses
  ☐ Cancer-inhibitory medication (medication used during chemotherapy)
  ☐ Pesticides
  ☐ Heavy metals and/or metallic compounds (for example cadmium, mercury, lead, mangenese and chromium)
  ☐ Other, namely……………………………………………………
☐ I don't know

<u>B. Spare time</u>

16. Do you currently participate in a sport while pregnant?

☐ No, and I don't normally participate in sports either
☐ No, I stopped participating in sports as soon as I realised I was pregnant
☐ Yes, namely: (fill in which sport)…………………………………………………………
  ☐ Less than two hours a week
  ☐ More than two hours a week

17. Are you currently spending less time on your hobbies or sports because of your pregnancy??

☐ No
☐ Yes

C. Domestic situation

18. Who is currently doing the housekeeping?

- ☐ You are largely doing it yourself
- ☐ You are doing it together with your partner/someone else
- ☐ Your partner/someone else is doing the most of it

19. Do you have a household help?

- ☐ No
- ☐ Yes, for …………. hours a week (on average)

End of the questionnaire. Thank you for completing the form!

Space for remarks:

PROPELLOR - Prevention of premature birth

Questionnaire 3

Completed between 30 to 34 weeks of gestation

Questionnaire "Working conditions and workload"

Please note: this questionnaire is about the past 10 weeks (since you filled in part 2 of the questionnaire)!

A. Work (outdoors):

3. What is your current work status?

☐ I am still working
☐ I am working part-time due to complaints/illness
☐ I am no longer working due to complaints/illness
☐ I am on pregnancy leave
☐ I do not have a paid job → ***Please continue with part B***

4. Have you received any information when you informed your employer of your pregnancy?

☐ No
☐ No, I am an independent entrepreneur
☐ Yes, about **(more than one answer possible)**
  ☐ Pregnancy and maternity leave
  ☐ Combination breastfeeding/expressing and work
  ☐ extra rest periods
  ☐ working conditions and any possible adjustments to the work

3. Have adaptions been made to your work <u>because you are currently pregnant</u>?

☐ No → ***continue with question 6***
☐ Yes, namely…… ***(more than one answer possible)***
  ☐ Less physically taxing work
  ☐ Less standing or walking
  ☐ Lifting or carrying less or less often
  ☐ Slower work pace
  ☐ Less work
  ☐ Other working hours
  ☐ Less hours a day
  ☐ No more night shifts
  ☐ More opportunity to plan the work at your own discretion
  ☐ More working from home
  ☐ Other adjustments, namely ……………………………………………………………………

These adjustments have been in effect since …………. weeks of pregnancy

4. If adjustments have been made to your work, did you feel the need for these adjustments?

☐ No

☐ Yes

5. Who advised you to adjust your work because of your pregnancy? ***(more than one answer possible)***

☐ Obstetrician

☐ Gynaecologist

☐ Company doctor

☐ General practitioner

☐ Manager

☐ Staff advisor

☐ Yourself (at your own initiative)

☐ Other, namely …..

6. Have you had an increase in complaints that limit you in your work <u>because of your pregnancy</u>?

☐ No

☐ Yes, namely…… ***(more than one answer possible)***

- ☐ Complaints of fatigue
- ☐ Headaches
- ☐ Complaints of pain in the back, pelvis and/or legs
- ☐ Nausea/vomiting
- ☐ Stomach aches
- ☐ Contractions
- ☐ Other complaints, namely………………………………………………………………………

If you are currently no longer working **→ Please continue with part B and then C**

7.a. How many hours per week do you currently work on average? ……… hours a week

7.b. How many days per week do you work on average? ……… days a week

8. Do you work in irregular shifts?

☐ No

☐ Yes

How many of your working hours do you on average spend on these shifts **<u>a week</u>**?

- ■ ………. hours a week in day shifts
- ■ ………. hours a week in evening shifts (until 23:00)
- ■ ………. hours a week in night shifts

9a. During your work, how many hours a day do you have to:

- ☐ Walk ...... hours a day
- ☐ Stand ...... hours a day
- ☐ Sit ...... hours a day

9b. If you have to sit, do you have the opportunity to sometimes get up and stand or walk for a while?

- ☐ Always
- ☐ Sometimes
- ☐ Rarely
- ☐ Never

9c. If you have to walk or stand, do you have the opportunity to sometimes sit down for a while:

- ☐ Always
- ☐ Sometimes
- ☐ Rarely
- ☐ Never

10a. Do you have to lift and/or carry things during your work?

- ☐ No → ***continue with question 11***
- ☐ Yes → ***continue with question 10b***

10b. During my work I lift or carry:

- ☐ People
- ☐ Animals
- ☐ Loads

10c. In the table below you can fill in how much weight you lift and how often this occurs. More than one answer is possible. If you are lifting loads or people heavier than 20kg, please fill in how much kg they weigh on average.

	Less than 5 kg	5-10 kg	10-20 kg	More than 20 kg
1-10 times a day (once an hour)	☐	☐	☐	☐ ....kg
10-25 times a day (2 or 3 times an hour)	☐	☐	☐	☐ ....kg
25-50 times a day (4 to 6 times an hour)	☐	☐	☐	☐ ....kg
50-100 times a day (7 to 12 times an hour)	☐	☐	☐	☐ ....kg
More than 100 times a day (13 times an hour)	☐	☐	☐	☐ .... kg

11. For the next couple of questions, please indicate every time <u>how often</u> certain things occur at the moment. You can choose between the following answers:
never, sometimes, often, regularly.
Try not to skip any questions.

		never	sometimes	regularly	often
a.	Do you have to bend over during work?	☐	☐	☐	☐
b.	Do you have to squat during work?	☐	☐	☐	☐
c.	Do you have to reach out high with your arms during work?	☐	☐	☐	☐
d.	Do you have to make the same repetitive motion a lot during your work?	☐	☐	☐	☐
e.	Do you think your work is very physically demanding?	☐	☐	☐	☐
f.	Does your work require physical strength?	☐	☐	☐	☐
g.	Do you work in uncomfortable or strenuous postures?	☐	☐	☐	☐
h.	Do you work at a strenuous machine or assembly line?	☐	☐	☐	☐

12. During your work, are you often exposed to one of the factors below?

		never	sometimes	regularly	often
a.	High temperatures/heat	☐	☐	☐	☐
b.	Low temperatures/coldness	☐	☐	☐	☐
c.	Noise	☐	☐	☐	☐
d.	Body vibrations	☐	☐	☐	☐
e.	Overpressure	☐	☐	☐	☐

13. For the next couple of questions, please indicate every time <u>how often</u> certain things occur at this moment. You can choose between the following answers:
never, sometimes, often, always. Try not to skip any questions.

		never	sometimes	often	always
a.	Do you have problems with the pace of work?	☐	☐	☐	☐
b.	Do you have problems with the pressure of work?	☐	☐	☐	☐
c.	Would you like to take things a little easier in your work?	☐	☐	☐	☐
d.	Are you free in the performance of your tasks?	☐	☐	☐	☐
e.	Do you have any influence on the pace of work?	☐	☐	☐	☐
f.	Can you plan your work yourself?	☐	☐	☐	☐
g.	Do you experience sufficient support from your manager?	☐	☐	☐	☐
h.	Do you experience sufficient support from your colleagues?	☐	☐	☐	☐
i.	Do you enjoy your work?	☐	☐	☐	☐
j.	Are you satisfied with your work?	☐	☐	☐	☐

14. Is there a chance for you to contact an infectious disease at your work?

- ☐ No
- ☐ Yes; I regularly work with: ***(more than one answer possible):***
  - ☐ Ill and/or small children
  - ☐ Ill adults
  - ☐ Animals
  - ☐ Nature, in forests or public gardens
  - ☐ Raw meat
  - ☐ Waste or waste water
  - ☐ Blood or other bodily fluids (also via waste)
  - ☐ Stools (also via waste)

15. Do you come into contact with chemical substances during your work?

- ☐ No
- ☐ Yes, with: ***(more than one answer possible)***
  - ☐ Cleaning supplies
  - ☐ Solvents (for example in paint, lacquer, glue, detergents and ink)
  - ☐ Anaesthetic gasses
  - ☐ Cancer-inhibitory medication (medication used during chemotherapy)
  - ☐ Pesticides
  - ☐ Heavy metals and/or metallic compounds (for example cadmium, mercury, lead, mangenese and chromium)
  - ☐ Other, namely………………………………………………………
- ☐ I don’t know

B. Spare time

16. Do you currently participate in a sport while pregnant?

☐ No, and I don't normally participate in sports either
☐ No, I stopped participating in sports as soon as I realised I was pregnant
☐ Yes, namely: (fill in which sport)………………………………………………………………
 ☐ Less than two hours a week
 ☐ More than two hours a week

17. Are you currently spending less time on your hobbies or sports because of your pregnancy?

☐ No
☐ Yes

C. Domestic situation

18. Who is currently doing the housekeeping?

☐ You are largely doing it yourself
☐ You are doing it together with your partner/someone else
☐ Your partner/someone else is doing the most of it

19. Do you have a household help?

☐ No
☐ Yes, for …………. hours a week (on average)

End of the questionnaire. Thank you for completing the form!

Space for remarks:
