# Supplementary material for: Risk factors for spontaneous preterm birth among healthy nulliparous pregnant women in the Netherlands, a prospective cohort study
Source: Health Sci Rep. 2022 May 24;5(3):e585. doi: 10.1002/hsr2.585 (PMC9127744; doi:10.1002/hsr2.585)
Supplement: Supplementary file 3 — Supplementary information. [file HSR2-5-e585-s004.docx]

**SUPPLEMENTAL METHODS**

**Questionnaires**

The first questionnaire, completed at inclusion, included socio-demographics, general health, current pregnancy, obstetric history, obstetric family history, current working conditions, leisure, household characteristics and possible domestic violence.

Questions from a validated questionnaire about psychosocial job strain and physically demanding work were used, supplemented with questions about other working conditions (e.g. (irregular) working times, chemical, biological and physical factors (noise, climate, body vibrations).(1) The questions on possible abuse and intimate partner violence were based questionnaires used in the Prevalence of abuse and intimate partner violence surgical evaluation (PRAISE) study.(2) Questionnaire 2 and 3 concerned (adjustment of) working conditions, leisure and household characteristics in the second and third trimester. Questionnaires were either completed on paper or online via a website developed for the study. The participating midwifery practice provided reminders for the participants for completing the paper questionnaires. Reminders for completing the online questionnaire were sent by email via the website.

**Bio-samples**

A self-administered vaginal swab was collected at inclusion. The vaginal swab was immediately stored at -20⁰C until transfer to the central storage facility at -80⁰C. Blood samples, consisting of a buffy coat, serum and plasma sample, were collected during the routine antenatal blood workup in the first trimester. The collected tubes were immediately frozen at -80⁰C.

**Sample size**

The study was initially aimed at developing a prediction model for spontaneous preterm birth using clinical risk factors. To evaluate 20 variables with an expected incidence of 5% in this population required 4000 participants. The funding agent terminated the study due to low recruitment rate, after reaching 9.1% of the estimated sample size.

1. Vrijkotte TG, van der Wal MF, van Eijsden M, Bonsel GJ. First-trimester working conditions and birthweight: a prospective cohort study. American journal of public health. 2009;99(8):1409-16.

2. Investigators P, Sprague S, Bhandari M, Della Rocca GJ, Goslings JC, Poolman RW, et al. Prevalence of abuse and intimate partner violence surgical evaluation (PRAISE) in orthopaedic fracture clinics: a multinational prevalence study. Lancet. 2013;382(9895):866-76.
